# Efficacy of a new technique – INtubate-RECruit-SURfactant-Extubate – “IN-REC-SUR-E” – in preterm neonates with respiratory distress syndrome: study protocol for a randomized controlled trial

**DOI:** 10.1186/s13063-016-1498-7

**Published:** 2016-08-18

**Authors:** Giovanni Vento, Roberta Pastorino, Luca Boni, Francesco Cota, Virgilio Carnielli, Filip Cools, Carlo Dani, Fabio Mosca, Jane Pillow, Graeme Polglase, Paolo Tagliabue, Anton H. van Kaam, Maria Luisa Ventura, Milena Tana, Chiara Tirone, Claudia Aurilia, Alessandra Lio, Cinzia Ricci, Alessandro Gambacorta, Chiara Consigli, Danila D’Onofrio, Camilla Gizzi, Luca Massenzi, Viviana Cardilli, Alessandra Casati, Roberto Bottino, Federica Pontiggia, Elena Ciarmoli, Stefano Martinelli, Laura Ilardi, Mariarosa Colnaghi, Piero Giuseppe Matassa, Valentina Vendettuoli, Paolo Villani, Francesca Fusco, Diego Gazzolo, Alberto Ricotti, Federica Ferrero, Ilaria Stasi, Rosario Magaldi, Gianfranco Maffei, Giuseppe Presta, Roberto Perniola, Francesco Messina, Giovanna Montesano, Chiara Poggi, Lucio Giordano, Enza Roma, Carolina Grassia, Gaetano Ausanio, Fabrizio Sandri, Giovanna Mescoli, Francesco Giura, Giampaolo Garani, Agostina Solinas, Maria Lucente, Gabriella Nigro, Antonello Del Vecchio, Flavia Petrillo, Luigi Orfeo, Lidia Grappone, Lorenzo Quartulli, Antonio Scorrano, Hubert Messner, Alex Staffler, Giancarlo Gargano, Eleonora Balestri, Stefano Nobile, Caterina Cacace, Valerio Meli, Sara Dallaglio, Betta Pasqua, Loretta Mattia, Eloisa Gitto, Marcello Vitaliti, Maria Paola Re, Stefania Vedovato, Alessandra Grison, Alberto Berardi, Francesco Torcetta, Isotta Guidotti, Sandra di Fabio, Eugenia Maranella, Isabella Mondello, Stefano Visentin, Francesca Tormena

**Affiliations:** 1 Division of Neonatology, Department for the Protection of Women’s Health and the Nascent Life, Child and Adolescent, Policlinico A. Gemelli – Università Cattolica del Sacro Cuore, Largo A. Gemelli 8, 00168 Rome, Italy; 2 Section of Hygiene, Institute of Public Health, Università Cattolica del Sacro Cuore, Largo Francesco Vito 1, 00168 Rome, Italy; 3 Clinical Trials Coordinating Center of Istituto Toscano Tumori, Department of Oncology, Careggi University Hospital, Largo Brambilla 3, 50134 Florence, Italy; 4 Division of Neonatology, Department of Clinical Sciences, Polytechnic University of Marche and Azienda Ospedaliero Universitaria Ospedali Riuniti, Ancona, Italy; 5 Department of Neonatology, Universitair Ziekenhuis Brussel, Laarbeeklaan 101, 1090 Brussels, Belgium; 6 Department of Surgical and Medical Critical Care, Section of Neonatology, Careggi University Hospital, Viale Morgagni 85, 50141 Florence, Italy; 7 Department of Clinical Sciences and Community Health, University of Milan-Fondazione IRCCS Cà Granda Ospedale Maggiore Policlinico, Via Della Commenda 12, 20122 Milan, Italy; 8 School of Anatomy, Physiology and Human Biology, University of Western Australia, Crawley, WA Australia; 9 The Ritchie Centre Hudson Institute of Medical Research and Department of Obstetrics and Gynaecology, Monash University, Clayton, 3168 VIC Australia; 10 Fondazione MBBM - Ospedale San Gerardo, Monza, Italy; 11 Department of Neonatology, Emma Children’s Hospital, Academic Medical Center, Amsterdam, The Netherlands; 12 Ospedale San Pietro Fatebenefratelli, Rome, Italy; 13 Ospedale S. Giovanni Calibita Fatebenefratelli Isola Tiberina, Rome, Italy; 14 Università di Roma “La Sapienza”/Policlinico Umberto I, Rome, Italy; 15 Ospedale Belcolle, Viterbo, Italy; 16 Fondazione Poliambulanza, Brescia, Italy; 17 Ospedale Niguarda Cà Granda, Milan, Italy; 18 Azienda Ospedaliera Carlo Poma, Mantova, Italy; 19 Azienda Ospedaliera Nazionale SS. Antonio e Biagio e Cesare Arrigo, Alessandria, Italy; 20 Azienda Ospedaliero Universitaria Maggiore della Carità, Novara, Italy; 21 Azienda Ospedaliero-Universitaria Ospedali Riuniti, Foggia, Italy; 22 Azienda Ospedaliera Vito Fazzi, Lecce, Italy; 23 Ospedale Evangelico Villa Betania, Naples, Italy; 24 Careggi University Hospital, Florence, Italy; 25 Casa di Cura Pineta Grande, Castelvolturno (CE), Italy; 26 Azienda Ospedaliera Sant’ Anna e San Sebastiano, Caserta, Italy; 27 Ospedale Maggiore, Bologna, Italy; 28 Azienda Ospedaliero-Universitaria Arcispedale S. Anna, Ferrara, Italy; 29 Azienda Ospedaliera di Cosenza, Cosenza, Italy; 30 Ospedale Di Venere, Bari, Italy; 31 A.O.R.N. “G. RUMMO”, Benevento, Italy; 32 Ospedale Panico, Tricase (LE), Italy; 33 Ospedale di Bolzano, Bolzano, Italy; 34 Azienda Ospedaliera Arcispedale S.Maria Nuova di Reggio Emilia/IRCCS, Reggio Emilia, Italy; 35 Polytechnic University of Marche and Azienda Ospedaliero Universitaria Ospedali Riuniti, Ancona, Italy; 36 Ospedale Barone Romeo, Patti (ME), Italy; 37 Azienda Ospedaliera-Universitaria di Parma, Parma, Italy; 38 Azienda Ospedaliero-Universitaria Policlinico Vittorio Emanuele- PO G. Rodolico, Catania, Italy; 39 Azienda Ospedaliero-Universitaria Policlinico “G. Martino”, Messina, Italy; 40 Azienda Ospedaliera di rilievo nazionale e di alta specializzazione Arnas Civico, Palermo, Italy; 41 Ospedale San Bortolo, Vicenza, Italy; 42 Azienda Ospedaliera-Universitaria di Modena, Modena, Italy; 43 Ospedale San Salvatore, L’Aquila, Italy; 44 Azienda ospedaliera “Bianchi-Melacrino-Morelli”, Reggio Calabria, Italy; 45 Ospedale Santa Maria di Ca’ Foncello di Treviso, Treviso, Italy

**Keywords:** Preterm infants, Lung recruitment, HFOV, INSURE

## Abstract

**Background:**

Although beneficial in clinical practice, the INtubate-SURfactant-Extubate (IN-SUR-E) method is not successful in all preterm neonates with respiratory distress syndrome, with a reported failure rate ranging from 19 to 69 %. One of the possible mechanisms responsible for the unsuccessful IN-SUR-E method, requiring subsequent re-intubation and mechanical ventilation, is the inability of the preterm lung to achieve and maintain an “optimal” functional residual capacity. The importance of lung recruitment before surfactant administration has been demonstrated in animal studies showing that recruitment leads to a more homogeneous surfactant distribution within the lungs. Therefore, the aim of this study is to compare the application of a recruitment maneuver using the high-frequency oscillatory ventilation (HFOV) modality just before the surfactant administration followed by rapid extubation (INtubate-RECruit-SURfactant-Extubate: IN-REC-SUR-E) with IN-SUR-E alone in spontaneously breathing preterm infants requiring nasal continuous positive airway pressure (nCPAP) as initial respiratory support and reaching pre-defined CPAP failure criteria.

**Methods/design:**

In this study, 206 spontaneously breathing infants born at 24^+0^–27^+6^ weeks’ gestation and failing nCPAP during the first 24 h of life, will be randomized to receive an HFOV recruitment maneuver (IN-REC-SUR-E) or no recruitment maneuver (IN-SUR-E) just prior to surfactant administration followed by prompt extubation. The primary outcome is the need for mechanical ventilation within the first 3 days of life. Infants in both groups will be considered to have reached the primary outcome when they are not extubated within 30 min after surfactant administration or when they meet the nCPAP failure criteria after extubation.

**Discussion:**

From all available data no definitive evidence exists about a positive effect of recruitment before surfactant instillation, but a rationale exists for testing the following hypothesis: a lung recruitment maneuver performed with a step-by-step Continuous Distending Pressure increase during High-Frequency Oscillatory Ventilation (and not with a sustained inflation) could have a positive effects in terms of improved surfactant distribution and consequent its major efficacy in preterm newborns with respiratory distress syndrome. This represents our challenge.

**Trial registration:**

ClinicalTrials.gov identifier: NCT02482766. Registered on 1 June 2015.

## Background

The pathogenesis of bronchopulmonary dysplasia (BPD) in preterm infants is multifactorial, but the role of ventilator-induced lung injury (VILI) is very important [1]. Although the respiratory support of preterm infants with respiratory distress syndrome (RDS) has improved and new modes of mechanical ventilation have been developed, the incidence of BPD has remained unchanged. Avoiding invasive mechanical ventilation by starting non-invasive respiratory support, such as continuous positive airway pressure (CPAP), in the delivery room is considered an important first step in reducing VILI and BPD. In case invasive mechanical ventilation is necessary, the recruitment of collapsed alveoli, thereby creating an optimal functional residual capacity (FRC) and limiting exposure to large tidal volumes and repetitive “opening and closing” of the unstable alveoli, may be the most effective way to reduce the risk of VILI and subsequent BPD.

The findings from the COIN [2], SUPPORT [3], and VON DRM [4] trials – comparing Early CPAP versus standard care (intubate, surfactant, mechanical ventilation) – are remarkably consistent. No single trial was able to demonstrate a statistically significant difference in the risk of death or BPD when infants were managed initially with CPAP compared to mechanical ventilation. From the results of these three studies, it is clear that initial stabilization on CPAP, and provision of rescue surfactant only when necessary, is at least as beneficial, and quite possibly preferred, over the standard therapy of intubation of all infants at risk in the delivery room and needing subsequent support with mechanical ventilation. The Italian Neonatal Network report of 2012 showed that an initial nasal continuous positive airway pressure (nCPAP) approach was used in approximately 60 % of cases, while Dargaville et al. [5] have reported that 50 % of newborns with gestational age (GA) 25–28 weeks were initially managed with nCPAP, substantially lower than the 65–90 % reported in other studies [6, 7]. The percentage of CPAP failure in the newborns of 25–28 weeks’ gestation is 45 % in the Australian experience [5], higher than that reported by Ammari et al. (25 %) [6] and similar to that reported by De Jaegere et al. (50 %) [7] and in the COIN trial (46 %) [2]. Therefore, the optimal respiratory care of newborn infants with RDS may involve yet another choice.

As a potential alternative, the IN-SUR-E (INtubate-SURfactant-Extubate) approach [8] is very attractive. Recently, several studies have investigated the efficacy of combining non-invasive ventilation and surfactant, administered by transient intubation (IN-SUR-E). The findings from the CURPAP [9] and VON DRM [4] trials – comparing Early IN-SUR-E versus Early CPAP as initial stabilization – showed no differences in mortality, BPD or any other outcome. In the VON DRM trial 51 % of infants in the IN-SUR-E group were later intubated. Although beneficial in clinical practice, the IN-SUR-E method cannot be universally applied to all preterm neonates with RDS with a reported failure rate ranging from 19 to 69 % [10, 11]. The reported risk factors predicting a failure of IN-SUR-E are low birth weight, low gestational age, the severity of initial respiratory disease, and a low hemoglobin concentration prior to surfactant administration [10, 12, 13]. No randomized controlled trials have directly evaluated the efficacy of IN-SUR-E in extremely preterm neonates (above 28 weeks’ gestation) [8]. Nevertheless, the data of the “Sustained Lung Inflation (SLI) study” [14] recently conducted on infants with GA 25^+0^–28^+6^ weeks showed that nCPAP failure and the need for MV at 72 h of life was 53 % in the SLI group and 65 % in the control group (only nCPAP). In this study surfactant was preferably administered with the IN-SUR-E approach at a FiO_2_ threshold of 0.40, suggesting that at least 50 % of 25–28 weeks’ gestation infants need mechanical ventilation in the first 72 h of life, despite receiving a SLI maneuver and IN-SUR-E treatment for CPAP failure. One of the possible mechanisms responsible for the unsuccessful IN-SUR-E, requiring subsequent re-intubation and mechanical ventilation is the inability of the preterm lung with RDS to achieve and maintain an “optimal” FRC. Prophylactic or early rescue surfactant administration before alveolar recruitment probably results in an uneven surfactant distribution to already-open alveoli, thus resulting in poor clinical response to the first surfactant dose. The immediate positive effects of surfactant on oxygenation are primarily attributable to the stabilization of already-open alveoli, and not due to recruitment of collapsed ones. The importance of lung recruitment before surfactant administration has been demonstrated by Krause and colleagues [15] in a piglet model of lung injury where a volume recruitment maneuver by means of moderately increased tidal volumes or increased PEEP or both, improved gas exchange and lung function owing to more homogeneous surfactant distribution within the lungs. Volume recruitment was proven by improved compliance and increased FRC in all the intervention groups. We, therefore, seek to compare the application of a recruitment maneuver (in HFOV modality) just before the surfactant administration, followed by rapid extubation (INtubate-RECruit-SURfactant-Extubate: IN-REC-SUR-E) with IN-SUR-E alone in spontaneously breathing preterm infants requiring nCPAP as initial respiratory support and reaching pre-defined CPAP failure criteria, for evaluating its effectiveness in decreasing the need of MV and improving respiratory outcome.

### Trial hypothesis

The primary hypothesis of this study is that there will be a reduction in the need of mechanical ventilation in the first 72 h of life (excluding the transient tracheal intubation performed for surfactant administration and the mechanical ventilation administered for lung recruitment) in spontaneously breathing infants born at 24^+0^–27^+6^ weeks’ gestation and failing nCPAP during the first 24 h of life who received an HFOV recruitment maneuver (IN-REC-SUR-E) compared to those receiving no recruitment maneuver (IN-SUR-E) just prior to surfactant administration followed by prompt extubation.

## Methods/design

### Study design

This will be an unblinded multi-center randomized trial of IN-REC-SUR-E versus IN-SUR-E in infants born at 24^+0^–27^+6^ weeks’ gestation.

### Participating centres

The following Italian centres are actively recruiting for the trial:

Policlinico Gemelli, Rome; S. Pietro Fatebenefratelli, Rome; Fatebenefratelli-Isola Tiberina, Rome; Policlinico Umberto I, Rome; Bel Colle Hospital, Viterbo; Fondazione Poliambulanza, Brescia; Fondazione MBBM – Ospedale San Gerardo, Monza; Niguarda Hospital, Milan; Fondazione IRCCS Cà Granda Ospedale Maggiore Policlinico, University Milan, Milan; AO Carlo Poma, Mantova; SS Antonio e Biagio e Cesare Arrigo Hospital, Alessandria; Maggiore Hospital, Novara; AOU Ospedali Riuniti, Foggia; AO Vito Fazzi, Lecce; Villa Betania, Naples; Careggi University, Florence; Pineta Grande, Castelvolturno; AO S. Anna-S. Sebastiano, Caserta; Maggiore Hospital, Bologna; AOU, Ferrara; AO, Cosenza; Di Venere Hospital, Bari; AO G. Rummo, Benevento; Panico Hospital, Tricase; Bolzano Hospital; Arcispedale Santa Maria Nuova, Reggio Emilia; Salesi Hospital, Ancona; Barone Romeo Hospital, Patti; AOU Parma; AOU Policlinico Vittorio Emanuele-Gaspare Rodolico, Catania; Messina University; Arnas Civico Hospital, Palermo; San Bortolo Hospital, Vicenza; AOU Policlinico, Modena; San Salvatore Hospital, L’Aquila; AO Bianchi-Melacrino-Morelli, Reggio Calabria; AO Cà Foncello, Treviso.

Based on the last Italian survey on behalf of the Neonatal Pneumology Study Group (not yet published), most of the participant centers use HFOV “open lung strategy” as a first line ventilatory management of preterm infants with RDS. The centers which do not use this approach (but use HFOV as a “rescue” therapy), have experienced the “open lung” HFOV maneuver before starting the study, after an on-site training.

We estimate that the participating centres will have a minimum of 5 and a maximum of 20 eligible patients during the study period with 80 % assumed consent rate.

### Inclusion criteria

Infants satisfying the following inclusion criteria will be eligible to participate:

1. In-born at 24^+0^–27^+6^
2. spontaneously breathing at birth but requiring respiratory support (CPAP or O_2_) at 5 min of life
3. Parental consent has been obtained
4. Failing nCPAP during the first 24 h of life

### Exclusion criteria

1. Severe birth asphyxia or a 5-min Apgar score <3
2. Endotracheal intubation in the delivery room for resuscitation or insufficient respiratory drive according to AAP guidelines [16].
3. Prolonged premature rupture of membranes (PROM) for more than 3 weeks
4. Presence of major congenital malformations
5. Hydrops fetalis
6. Inherited disorders of metabolism

### Sample size

We hypothesized that a recruitment maneuver (in HFOV modality) before surfactant administration might decrease of the need of subsequent mechanical ventilation during the first 72 h of life from 50 % [2, 5, 7] to 30 %. We calculated that 103 newborns must be enrolled in each group to detect this difference as statistically significant with 80 % power at the 0.05 alpha level using the two-sided Pearson’s chi-square test.

### Randomisation

Infants will be allocated to one of the two treatment groups in a 1:1 ratio according to the minimization method, using an interactive web-based electronic system. Randomization will be stratified by center and gestational age (24^+0^–25^+6^ weeks or 26^+0^–27^+6^ weeks). A monthly accrual report about the study will be sent to the participating centers.

### Blinding

The study will not be blinded, and the staff performing the study also will take subsequent care of the infants.

### Management in the delivery room

Positive pressure with a neonatal mask and a T-piece system (Neopuff Infant Resuscitator ®, Fisher and Paykel, Auckland, New Zealand) will be used to stabilize the newborns after birth. All the neonates will receive one (or two) SLI maneuver(s) (25 cmH_2_O for 10–15 s) [14] and will be transferred to the NICU on nCPAP (6 cmH_2_O). If necessary, infants will start mechanical ventilation in agreement with the American Academy of Pediatrics’ guidelines on neonatal resuscitation [16]. In this latter case the babies will be excluded from the study (see Exclusion criteria section, page 4).

### CPAP failure criteria

In the NICU, nCPAP will be given through nasal prongs/mask using the standard method of the single centre (ventilator, flow-dependent system) with an initial pressure of 6 to 7 cmH_2_O, in all infants. CPAP failure is defined if they met any of the following criteria: FiO_2_ ≥ 0.30 on nCPAP [17] to maintain a SpO_2_ of 87–94 % [18] for at least 30 min unless rapid clinical deterioration has occurred; respiratory acidosis defined as pCO_2_ > 65 mmHg (8.5 kPa) and pH <7.20 on an arterial or capillary blood gas sample; apnea defined as more than four episodes of apnea per hour or more than two episodes of apnea per hour when ventilation with bag and mask will be required.

### HFOV recruitment maneuver

Infants in the IN-REC-SUR-E group will undergo the following approach: after intubation, HFOV will be delivered with the ventilator available in each NICU. The following initial ventilator setting will be advocated: continuous distending pressure (CDP): 8 cmH_2_O; frequency: 10–15 Hz; ∆P: 15 cmH_2_O or amplitude 30 % eventually increased – chest to be “visibly vibrating”; I:E 1:2. ΔP (or amplitude) first and/or frequency will subsequently be adjusted to achieve a tidal volume (V_T_) of 1.5–2 ml/kg and/or to maintain the transcutaneous partial carbon dioxide pressure (TcPCO_2_) between 40 and 60 mmHg (5.3 and 8.0 kPa). The infants will be subjected to an open lung ventilation strategy aiming to recruit and stabilize the majority of collapsed alveoli/sacculi, using oxygenation as an indirect parameter for lung volume. Optimal recruitment is defined as adequate oxygenation using a fraction of inspired oxygen (FiO_2_) of 0.25 or less. Starting at 8 cmH_2_O, the CDP will be increased stepwise (2 cmH_2_O every 2–3 min) as long as pulse oximetry (SpO_2_) improves. The FiO_2_ will be reduced stepwise, keeping SpO_2_ within the target range (87–94 %). The recruitment procedure will be stopped if oxygenation no longer improves or if the FiO_2_ is equal to or less than 0.25. The corresponding CDP will be called the opening pressure (CDP_O_). Next, the CDP will be reduced stepwise (1–2 cmH_2_O every 2–3 min) until the SpO_2_ deteriorates (by at least 2–3 points). The corresponding CDP will be called the closing pressure (CDP_C_). After a second recruitment maneuver at CDP_O_ for 2 min, the optimal CDP (CDP_OPT_) will be set 2 cmH_2_O above the CDP_C_ for at least 3 min [19]. A chest radiograph at this point is advised.

### Surfactant treatment

Infants in the IN-REC-SUR-E arm will undergo the following approach: as soon as possible after the recruitment maneuver (at CDP_OPT_) a dose of poractant alfa (Curosurf (Chiesi Farmaceutici, Parma, Italy)) of 200 mg/kg will be administered via a closed administration system in one to two aliquots (1–2 min). The tube position will be confirmed by auscultation. A temporary reduction of frequency may be necessary to increase the V_T_ up to 2.5 ml/kg for improving the surfactant spreading.

Infants in the IN-SUR-E arm will undergo the following approach: after intubation, a dose of poractant alfa (Curosurf (Chiesi Farmaceutici, Parma, Italy)) of 200 mg/kg will be administered via a closed administration system in one to two aliquots (1–2 min). The tube position will be confirmed by auscultation. During surfactant administration, infants will be manually ventilated to facilitate surfactant distribution. If necessary, mechanical ventilation with a peak inspiratory pressure (PIP) of 20–22 cmH_2_O, a PEEP of 5–6 cmH_2_O and a respiratory rate of 30–40 breaths/min will be subsequently started to achieve a V_T_ of 4–6 ml/kg and/or to maintain the transcutaneous partial carbon dioxide pressure (TcPCO_2_) between 40 and 60 mmHg (5.3 and 8.0 kPa).

After surfactant administration, the babies of both groups will be extubated within 30 min (if satisfactory respiratory drive is present) and will receive nCPAP (6–8 cmH_2_O) [20]. In case of insufficient respiratory drive, CDP and/or ΔP (in the babies of IN-REC-SUR-E arm) or PIP (in the IN-SUR-E arm) will be reduced until spontaneous respiratory activity is restored, to maintain the transcutaneous partial carbon dioxide pressure (TcPCO_2_) between 40 and 60 mmHg (5.3 and 8.0 kPa). Maintaining a FiO_2_ < 0.30 to obtain SpO_2_ values in the desired range (87–94 %) will drive the eventual reduction in the level of CPAP in the following days. The decision as to whether to begin bi-level nCPAP (BiPAP) or nasal intermittent MV (N-IMV) to prevent the need for re-intubation in infants of both groups will be up to the neonatologist on duty, and will be considered in the final analysis.

Infants of both groups can receive a subsequent dose of surfactant (100 mg/kg of poractant alfa) using the same method (IN-SUR-E or IN-REC-SUR-E) if they meet the CPAP failure criteria again during the following 12 to 24 h.

#### Caffeine

A loading dose of caffeine citrate (20 mg/kg) will be given immediately after admission to the NICU, followed by a daily maintenance dose of 5–10 mg/kg.

#### Analgesia-sedation

All neonates will receive pre-intubation medications to provide adequate analgesia and sedation while preserving spontaneous respiratory activity during the procedure of surfactant administration, according to the local protocols involving the use of opioids (fentanyl or remifentanil).

### Primary outcome measure

The primary outcome is the need for mechanical ventilation within the first 3 days of life and, therefore, we consider IN-REC-SUR-E or IN-SUR-E a success if mechanical ventilation is not required and a failure if the infant needs mechanical ventilation in the first 72 h. Infants in both groups will be considered to have reached the primary outcome also when they are not extubated within 30 min after surfactant administration or when they meet the nCPAP failure criteria after extubation.

### Secondary outcome measures

1. Duration (days) of nCPAP, need and duration of bi-level NCPAP (BiPAP) and nasal intermittent MV (N-IMV)
2. Need and duration (days) of conventional mechanical ventilation (synchronized intermittent mandatory ventilation (SIMV), synchronized intermittent positive pressure ventilation (SIPPV), pressure support ventilation (PSV) with or without volume guarantee (VG)) or high-frequency oscillatory ventilation (HFOV) with or without VG
3. Duration (days) of oxygen therapy and highest FiO_2_ level of oxygen concentration
4. Duration of hospitalization
5. Number of doses of surfactant during hospitalization
6. Occurrence of mild, moderate, and severe bronchopulmonary dysplasia (BPD) during hospitalization. The severity of BPD is categorized according to the consensus definition [21]: treatment with oxygen >21 % for at least 28 days, plus: mild BPD: breathing room air at 36 weeks post menstrual age (PMA) or discharge, whichever comes first; moderate BPD: need for more than 30 % oxygen at 36 weeks PMA or discharge, whichever comes first; severe BPD: need for at least 30 % oxygen and/or positive pressure (PPV or nCPAP) at 36 weeks PMA or discharge, whichever comes first
7. Time of being without any respiratory support

### Other collected data

The following data will be recorded for each infant: gestational age (GA), birth weight (BW), sex, Apgar score at 5 min, antenatal steroid treatment, prolonged PROM of more than 18 h, diagnosis of histological and/or clinical chorioamnionitis, type of delivery, clinical risk index for babies (CRIB) II score [22], occurrence of air leaks, pulmonary hemorrhage, patent ductus arteriosus (PDA) and need for surgical closure, timing of surfactant administration, grades 3–4 intraventricular hemorrhage (IVH) [23], periventricular leukomalacia (PVL) [24], higher than grade 2 retinopathy of prematurity (ROP) [25], necrotizing enterocolitis (NEC) [26], sepsis defined as a positive blood culture or suggestive clinical and laboratory findings leading to treatment with antibiotics for at least 7 days despite the absence of a positive blood culture, length of stay in the NICU, use of systemic postnatal steroids, and mortality. The discharge from NICU will be performed when infants will not need respiratory assistance other than using oxygen therapy and central venous catheter insertion.

### Data collection

All collected data can be obtained from the clinical records. They will be reported in electronic data sheets designed for this study. Data will be entered by the local principal investigator on a web-based electronic case record form. Access to the form will be password-protected and participants will be identified by trial number only. Clinical information will be collected at the following times:

1. At trial entry: information on eligibility; background information and randomization
2. Following randomisation: all data above listed in “Primary outcome measure,” “Secondary outcome measure,” and “Other collected data” sections

Further information will be collected on expected serious adverse events.

In terms of the PICOT format, our study can be described as follows: P: preterm infants of 24-27 weeks failing nCPAP during the first 24 h of life; I: surfactant administered during HFOV with optimal lung volume strategy; C: surfactant administration via INSURE method using conventional positive pressure ventilation; O: need for mechanical ventilation; T: study entry until 72 h of life.

### Statistical analysis

The primary efficacy analysis will be conducted on an intention-to-treat basis. An as-treated analysis will also be implemented for secondary analyses if a large number of infants do not end up getting the treatment to which they were assigned. Clinical characteristics of infants in the “IN-REC-SUR-E” and “IN-SUR-E” groups will be described using mean values and standard deviation, median value and range, or rate and percentage. Univariate statistical analysis will be performed using the Student’s *t* test for parametric continuous variables, the Wilcoxon rank-sum test for non-parametric continuous variables, and Fisher’s exact test for categorical variables. A *p* value <0.05 will be considered statistically significant. Then, treatment arm and clinical characteristics which are most likely associated with the need for mechanical ventilation (gestational age, birth weight, antenatal steroids, CRIB score) will be included in a multiple logistic regression analysis to assess their independent role in predicting the clinical outcome. Effect estimates will be expressed as odds ratios (ORs) with maximum likelihood-based 95 % confidence limits. An interim analysis will be planned when 50 infants will be enrolled in each arm. The analysis will intend to compare treatment arms with respect to efficacy, safety, futility, assessment of difficulties with patient enrollment, and, if necessary, a sample size adjustment. In the interim analysis for safety the pre-specified stopping rules are: a mortality rate more than 40 %, a rate of severe IVH more than 30 % and a pneumothorax rate of more than 10 %. The interim-analysis will be performed by an independent statistician, blinded for the treatment allocation. The statistician will report to the independent Data and Safety Monitoring Board (DSMB). The DSMB will have unblinded access to all data and will discuss the results of the interim-analysis with the Steering Committee in a joint meeting. The Steering Committee will decide on the continuation of the trial and will report to the central Ethics Committee.

#### Duration of study

In this study, 206 infants will be recruited. The trial will terminate when the last recruited infant is discharged from hospital, or dies.

### Quality control and quality assurance procedures

#### Compliance to protocol

Compliance will be defined as full adherence to protocol. Compliance with the protocol will be ensured by a number of procedures as described below.

#### Site set-up

Local principal investigators are required to participate in preparatory meetings in which details of study protocol, data collection, “IN-REC-SUR-E” and “IN-SUR-E” procedures will be accurately discussed. All centers will receive detailed written instruction on web-based data recording, and, to solve possible difficulties, it will be possible to contact the Clinical Trials Coordinating Center (Policlinico A. Gemelli, Rome). Moreover, it has been ascertained that the “IN-REC-SUR-E” procedure is followed similarly in all participating centers.

#### Data processing and monitoring

All study data will be:

1. Screened for out-of-range data, with cross-checks made for conflicting data within and between data collection forms by a data manager
2. Referred back to the relevant center for clarification in the event of missing items or uncertainty. A record of all discrepancies and resolutions will be kept by the data manger

The chief investigator and trial statistician will review the results generated for logic and for patterns or problems. Outlier data will be investigated.

#### Safety

Safety end-point measures will include incidence, severity, and causality of reported serious adverse events (SAEs), namely changes in the occurrence of the expected common prematurity complications and clinical laboratory test assessments, and the development of unexpected SAEs in this high-risk population. All SAEs will be followed until satisfactory resolution is achieved or until the investigator responsible for the care of the participant deems the event to be chronic or the patient to be stable. All expected and unexpected SAEs, whether or not they are attributable to the study intervention, will be reviewed by the local principal investigators to determine if there is a reasonable suspected causal relationship to the intervention. If the relationship is reasonable, SAEs will be reported to chief investigators who will report to the Ethics Committee and request all investigators to guaranty the safety of all participants.

#### Dissemination policy

The scientific integrity of the project requires that the data from all sites be analyzed study-wide and reported as such. All presentations and publications are expected to protect the integrity of the major objectives of the study; data that break the blind will not be presented prior to the release of mainline results. Recommendations as to the timing of presentation of such endpoint data and the meetings at which they might be presented will be given by the Steering Committee. Substantive contributions to the design, conduct, interpretation, and reporting of the clinical trial will be recognized through the granting of authorship on the final trial report. Individuals who fulfill authorship criteria will have final authority over the manuscript content.

## Discussion

Lung aeration and FRC achievement represents a key process for the fetal transition to newborn life. It depends on the spontaneous inspiratory efforts, which create transpulmonary pressure gradients [27], but also on subsequent recruitment maneuvers in newborns with RDS. Although surfactant replacement therapy is an established treatment in infant RDS, the optimum strategy for ventilatory management before, during, and after surfactant instillation remains to be elucidated. It is plausible to expect that the therapeutic benefits of exogenous surfactant therapy will be maximized by quickly and uniformly aerating the lung beforehand. Ingimarsson et al. examined whether a lung recruitment maneuver just before surfactant would affect the response to rescue treatment in immature lambs with established RDS [28]. Five pairs of preterm twin lambs with a gestational age of 127 days were delivered by cesarean section and supported by pressure-limited mechanical ventilation for 4 h. At 30 min of age, when all the lambs were in severe respiratory failure, they were treated with porcine surfactant, 200 mg/kg. One lamb in each pair was subjected to a lung recruitment maneuver consisting of five sustained inflations of 20 ml/kg just before surfactant instillation. At 10 min after surfactant treatment, all the lambs showed a large improvement in oxygenation and an increase in inspiratory capacity and static compliance. Except for a transiently better oxygenation after surfactant therapy in the recruitment group (*P* <0.05), there were no significant between-group differences in gas exchange or lung mechanics at any time point during the study. There was no difference in post-mortem intrapulmonary air volume or alveolar expansion in histologic lung sections between groups. This small study does not show any positive or negative effect of a lung recruitment maneuver on the response to rescue surfactant therapy in immature animals with RDS. To explore the impact of different surfactant doses and ventilation strategies on the efficacy of surfactant treatment and ventilator pressures in surfactant-depleted newborn piglets, van Kaam et al. found that the efficacy of surfactant treatment is less dose-dependent during open lung ventilation compared with conventional positive pressure ventilation [29]. Moreover, open lung ventilation preserves the response to delayed surfactant treatment in surfactant-deficient newborn piglets, in contrast to conventional ventilation. This sustained response is accompanied by an attenuation of secondary lung injury [30]. More recently Tingay et al. [31] described the interrelationship between antenatal steroids, exogenous surfactant, and two approaches to lung recruitment at birth on oxygenation and respiratory system compliance in preterm lambs. They found that the effectiveness of surfactant maybe enhanced using PEEP-based time-dependent recruitment strategies rather than approaches solely aimed at initial lung liquid clearance. In our previous randomized clinical trial comparing early HFOV versus SIMV, the babies in the HFOV arm receiving surfactant after a recruitment maneuver showed significantly higher values of dynamic respiratory compliance with respect to the SIMV-treated babies receiving surfactant without a recruitment maneuver [32]. Our hypothesis was that the atelectatic lung units were more adequately opened by an optimal volume HFOV strategy than by SIMV, suggesting there to be a recruitment of new alveolar units rather than stabilization and distension of small airways and alveolar spaces alone. From all the available data, no definitive evidence exists about a positive effect of recruitment before surfactant instillation, but a rationale exists for testing the following hypothesis: a lung recruitment maneuver performed with a step-by-step CDP increase during HFOV (and not with a sustained inflation) could have a positive effect in terms of improved surfactant distribution and consequently have major efficacy in preterm newborns with RDS. This represents our challenge.

There are some limitations in our study design: (1) surfactant replacement therapy involves instillation of a liquid-surfactant mixture directly into the lung airway tree. Thus, surfactant distribution and efficacy are not only affected by lung recruitment before its administration but also depend on the methodology used for delivery of the drug and on the post-administration ventilatory management (prevention of collapse due to inadequate weaning). In our study, different methods of surfactant delivery will be used between the two groups. In the group of infants submitted to a lung recruitment maneuver, surfactant will be delivered into the airways without disconnection from the high frequency ventilator. The control group will receive surfactant while manually ventilated. On the other hand we cannot recruit and stabilize the lung if we start positive pressure ventilation in the IN-REC-SUR-E group, just to recruit at the time of administration of surfactant and delivery of positive pressure ventilation before/after its use. Recruitment is a conceptual design usually applied from start to finish. The fact that during IN-SUR-E we conventionally ventilate the baby and during HFOV we do not is inherent to the design of the study. If we would ‘bag’ the baby, recruitment will be lost and we will lose the contrast between the groups (administer surfactant in recruited or non-recruited lung). It is more an “open lung ventilation” that is tested against conventional positive pressure ventilation. Open lung ventilation includes surfactant administration in a recruited lung and avoiding injury after administration. The difference in ventilation during and after surfactant treatment is part of optimal lung volume strategy. This makes our study more of a “comparative effectiveness research” comparing two distinct approaches that consist of several components, rather than a simple randomized controlled trial (RCT) comparing surfactant with and without a recruitment maneuver; (2) failure of CPAP therapy is multifactorial. Local experience with this therapy in the smallest patients and type of CPAP applied seems to play an important role in outcomes. Based on the results of a recent survey on the neonatal respiratory support strategies in the Italian NICUs [33], nCPAP is the most commonly used non-invasive mode of respiratory support, both in the acute and post-extubation phases of RDS. The most commonly used devices in delivering non-invasive respiratory support are the Infant Flow® System (56 %), mechanical ventilators (33 %), and bubble CPAP (16 %). All the 37 NICUs involved in the present study have considerable experience with non-invasive respiratory support. Even though there could be potentially different outcomes according to the different devices used to deliver non-invasive respiratory support pre and post extubation, this should not affect the differences between the two interventions in our study, because of the randomization; (3) some studies do show a reduction in intubation need when N-IMV is used after extubation. Even though there could be potentially different outcomes according to the different non-invasive respiratory strategies used after extubation, it is conceivable that clinicians will apply a specific non-invasive ventilatory support (CPAP or BiPAP or N-IMV) similarly in both groups. Although there might be differences between centers, it will be similar within centers and because we randomize on center, this will not affect the differences between the two groups.

## Trial status

The trial is currently recruiting study subjects.

## Abbreviations

BPD, bronchopulmonary dysplasia; CDP, continuous distending pressure; CPAP, continuous positive airway pressure; FRC, functional residual capacity; GA, gestational age; HFOV, high-frequency oscillatory ventilation; IN-REC-SUR-E, INtubate-RECruit-SURfactant-Extubate; IN-SUR-E, Intubate-Surfactant-Extubate; IVH, intraventricular hemorrhage; nCPAP, nasal continuous positive airway pressure; NEC, necrotizing enterocolitis; NICU, neonatal intensive care unit; PDA, patent ductus arteriosus; PMA, post menstrual age; PROM, premature rupture of membranes; PSV, pressure support ventilation; PVL, periventricular leukomalacia; RDS, respiratory distress syndrome; ROP, retinopathy of prematurity; SAE, serious adverse event; SIMV, synchronized intermittent mandatory ventilation; SIPPV, synchronized intermittent positive pressure ventilation; SLI, Sustained Lung Inflation; VILI, ventilator-induced lung injury
